
JOURNAL INFORMATION
==============================
NLM Title Abbreviation: ScientificWorldJournal
Journal ID: ScientificWorldJournal
NLM Journal ID: 101131163
Journal ID: TSWJ

The Scientific World Journal

ISSN: 2356-6140
EISSN: 1537-744X
Publisher: Wiley

ARTICLE INFORMATION
==============================
PMCID: PMC6139117
PMID: 30245598
DOI: 10.1100/tsw.2001.116
Article ID: 256269
Article version: 1
Subjects: Short Report

Mitochondrial Genes in Degenerative Disease and Aging

Wallace Douglas C.
Emory University School of Medicine, Atlanta, GA 30322, USA
Publication date: 2001
Electronic publication date: 2001 Oct 23
Volume: 1
First page: 83
Last page: 84
Copyright: Copyright © 2001 Douglas C. Wallace.
Copyright year: 2001
License: This is an open access article distributed under the Creative Commons Attribution License, which permits unrestricted use, distribution, and reproduction in any medium, provided the original work is properly cited.
License URL: https://creativecommons.org/licenses/by/3.0/

==============================
Miami *Nature Biotechnology* Short Reports
*TheScientificWorld* (2001) 1(S3), 83-84SR
ISSN 1532-2246; DOI 10.1100/TSW.2001.116

### MITOCHONDRIAL GENES IN DEGENERATIVE DISEASE AND AGING

Douglas C. Wallace

Emory University School of Medicine, Atlanta, GA 30322

It is becoming increasingly apparent that mitochondrial dysfunction is a central factor in degenerative diseases and aging.

**MITOCHONDRIAL PHYSIOLOGY.** One of the primary functions of the mitochondrion is the generation of cellular energy by the process oxidative phosphorylation (OXPHOS). OXPHOS encompasses the electron transport chain (ETC) consisting of NADH dehydrogenase (complex I), succinate dehydrogenase (complex II), the $bc_1$ complex (complex III) and cytochrome c oxidase (complex IV). Oxidation of NADH or succinate by the ETC generates an electrochemical gradient ($\Delta\psi$) across the mitochondrial inner membrane, which is utilized by the ATP synthase (complex V) to synthesis ATP. This ATP is exchanged for cytosolic ADP by the adenine nucleotide translocator (ANT). Inhibition of the ETC results in the accumulation of electrons in the beginning of the ETC, where they can be transferred directly to $O_2$ to give superoxide anion ($O_2^{\cdot -}$). Mitochondrial $O_2^{\cdot -}$ is converted to $H_2O_2$ by Mn superoxide dismutase (MnSOD), and $H_2O_2$ is converted to $H_2O$ by glutathione peroxidase (GPx1). The mitochondria are also the primary decision point for initiating apoptosis. This is mediated by the opening of the mitochondrial permeability transition pore (mtPTP), which couples the ANT in the inner membrane with porin (VDAC) in the outer membrane to the pro-apoptic Bax and anti-apoptic Bcl2. Increased mitochondrial $Ca^{++}$ or ROS and/or decreased $\Delta\psi$ or ATP tend to activate the mtPTP an initiate apoptosis (1).

**MITOCHONDRIAL DISEASES.** Mitochondrial diseases have been associated with both mtDNA and nuclear DNA (nDNA) mutations. MtDNA base substitution mutations resulting in maternally inherited diseases include both missense (protein) and protein synthesis (rRNA and tRNA) mutations. An example of a pathogenic mtDNA missense mutation is the ND6 gene mutation at nucleotide pair (np) 14459, which causes Leber’s hereditary optic neuropathy (LHON) and/or dystonia. The np 14459 mutation results in a marked complex I defect, and the segregation of the heteroplasmic mutation generates the two phenotypes along the same maternal lineage (2,3).

A relatively sever mitochondrial protein synthesis disease is caused by the np 8344 mutation in the tRNALys gene resulting in myoclonic epilepsy and ragged red fiber (MERRF) disease. Mitochondrial myopathy with ragged red muscle fibers (RRFs) and abnormal mitochondria is a common feature of severe mitochondrial disease. In MERRF families the severity of the phenotype correlates with the patient’s percentage of mutant mtDNAs and age. Indeed, a delayed onset and progressive course are common features of mtDNA diseases (4,5). The mildest mtDNA protein synthesis mutation is the np 4336 mutation in the tRNAGlu gene associated with late-onset Alzheimer (AD) and Parkinson Disease (PD) (6).

Degenerative diseases can also be caused by rearrangements in the mtDNA. Spontaneous mtDNA deletions often present with chronic progressive external ophthalmoplegia (CPEO) and mitochondrial myopathy, together with an array of other symptoms (7). Maternal-inherited mtDNA rearrangement diseases are more rare, but one family, manifesting adult-onset diabetes mellitus, sensory neural hearing loss, and stroke-like episodes, harbored a trimolecular heteroplasmy involving normal, 6.1 kb insertion, and reciprocal 10.4 kb deletion molecules (8,9).

Mitochondrial function also declines with age in the post-mitotic tissues of normal individuals. This is associated with the accumulation of somatic mtDNA rearrangement mutations in tissues such as skeletal muscle and brain (10-15). This same age-related accumulation of mtDNA rearrangements is seen in other multi-cellular animals including the mouse, where the accumulation of mtDNA damage is retarded by dietary restriction (16). Mitochondrial DNA point mutations also accumulate in post-mitotic tissues. Using protein nucleic acid (PNA)-directed PCR clamping to detect rare mutant molecules, the age-related control region, T414G, mutation (17) was found to accumulate in skeletal muscle after age 30, but not in brain even up to age 93 (18). However, using synaptosome fusions to transfer brain mtDNAs into cultured mtDNA-deficient ($\rho^O$) cells permitted us to recover mtDNAs with length variants in strings of Cs (19). This age-related accumulation of mtDNA mutations could exacerbate inherited mitochondrial defects and cause the delayed on-set and progressive course of mitochondrial disease.

**MOUSE MODELS AND THE PATHOPHYSIOLOGY OF MITOCHONDRIAL DISEASE.** To investigate the pathophysiology of mitochondrial disease, deleterious mitochondrial mutations have been introduced into mouse mtDNA and nDNA. The deleterious mtDNA mutation imparts resistance to the mitochondrial ribosomal inhibitor chloramphenicol (CAP). The nDNA mutations have inactivated the heart-muscle isoform of the ANT (Ant1), the GPx1, and the MnSOD.

The $CAP^R$ mtDNA mutation is the result of a T to C transition at np 2433 in the mtDNA 16S rRNA and causes partial respiratory deficiency. This mutation has been introduced into the mouse germline by fusing $CAP^R$ cytoplasts from mouse 501-1 cells to female embryonic stem (ES) cells, cured of their resident mtDNAs by treatment with rhodamine-6G (20). Chimeric mice generated from these $CAP^R$ ES cells developed bilateral nuclear cataracts and retinal pathology. Chimeric female mice transmitted the $CAP^R$ mtDNAs to their progeny and yielded both heteroplasmic and homoplasmic offspring. All $CAP^R$ neonates showed growth retardation and died in the neonatal period of mitochondrial myopathy and dilated cardiomyopathy (21).

To determine the importance of energy deficiency in mitochondrial disease, we have genetically-inactivation of the mouse Ant1 nDNA gene. Mice have two ANTs: ANT1, which is expressed at high levels in heart and skeletal muscle; and ANT2, which is expressed in all tissues except the skeletal muscle (22). As a consequence, Ant1 knockout mice derive almost no ATP from skeletal muscle mitochondria, reduced ATP from heart mitochondria, but normal ATP from liver mitochondria. Hence, the ANT1-deficient mice develop lacticacidosis, mitochondrial myopathy with sever fatigability and a hypertrophied cardiomyopathy that progresses to dilated cardiomyopathy (23).

Inhibition of ADP/ATP exchange results in the hyper-polarization of $\Delta\psi$, the inhibition of the ETC, and the generation of excess $O_2^{\cdot -}$. Consequently, mitochondria $H_2O_2$ production is elevated in heart, skeletal muscle, and brain. This increased ROS production is associated with the induction of MnSOD and GPx1, and an increase in mtDNA rearrangements (24).

The proliferation of mitochondria in the ANT-deficient skeletal muscle is similar to that seen in mitochondrial disease patients. This is associated with the coordinate induction of mtDNA and nDNA genes for mitochondrial biogenesis (25). Applying differential display (DD) (26) and hybridization to our 452 cDNA mitochondrial microarray (the Mitochip), we found that Ant1-deficient muscle undergoes an extensive up-regulation of genes for mitochondrial bioenergetics and biogenesis, ROS detoxification, and apoptosis.

To determine the importance of mitochondrial ROS toxicity in mitochondrial disease, we have genetically inactivated the GPx1 gene. Inactivation of GPx1 increases mitochondrial $H_2O_2$ production and resulted in a 20% reduction in weight and a one third reduction in the respiratory control ratio and the mitochondrial power output (27).

Genetic inactivation of the mitochondrial MnSOD would increase mitochondrial $O_2^{\cdot -}$ production. Homozygous mutant animals have a complete MnSOD-deficiency and die at approximately 8 days of age of a dilated cardiomyopathy with lipid accumulation in the liver (28), and inactivation of the iron-sulfur center containing enzymes of the mitochondria including complex I, II and mitochondrial aconitase (29). Heterozygous animals have a 50% reduction in MnSOD, which results in a life-long reduction in $\Delta\psi$, reduce ADP-stimulated respiration, increased endogenous respiration, and a marked sensitization of the mtPTP. In older animals, this leads to a marked increase in apoptosis. Analysis of Mitochip gene expression in older animals revealed the up-regulation of both anti-oxidant and apoptosis genes [Kokoszka, Coskun, Levy and Wallace].

Catalytic anti-oxidants such as MnTBAP can ameliorate the toxic effects of $O_2^{\cdot -}$. Treatment of MnSOD-deficient newborn mice rescued the cardiac defect, extending the mean life span to about 18 days. However, the MnTBAP does not cross the blood brain barrier, and the mice subsequently develop movement disorders and die of a spongiform encephalopathy (16). Treatment with other SOD mimetics (EUK8 and EUK134), which do cross the blood-brain barrier, further protected the animals. The relevance of these drugs to longevity was demonstrated by growing C. elegans in EUK134. The mev-1 mutant of C. elegans alters a complex II subunit, increases mitochondrial ROS production and shortens life-span. Growth of mev-1 in EUK134 restores normal life span. Furthermore, growth of wild type C. elegans in the drug extends their lifespan 50% (30). Therefore, mitochondrial ROS toxicity has been linked to longevity.

These data demonstrate that degenerative diseases and aging can be associated through a decline in mitochondrial function. In this model, an individual inherits a bioenergetic genotype that determines his initial level of mitochondrial bioenergetic capacity. As the individual ages, somatic mtDNA mutations accumulate, eroding his mitochondrial function. If the individual starts with a high energetic capacity, then many years are required before energy production become insufficient for normal tissue function. However, if the individual inherits an mitochondrial defect, then he starts at a lower energetic capacity and crosses energetic expression thresholds prematurely resulting in disease (1).

**REFERENCES.**

1. Wallace, D.C. (1999) Science 283, 1482-1488
2. Jun, A.S., Brown, M.D., and Wallace, D.C. (1994) Proc. Natl. Acad. Sci. U S A 91, 6206-6210
3. Jun, A.S. et al. (1996) Mol. Cell. Biol. 16, 771-777
4. Wallace, D.C. et al. (1988) Cell 55, 601-610
5. Shoffner, J.M. et al. (1990) Cell 61, 931-937
6. Shoffner, J.M. et al. (1993) Genomics 17, 171-184
7. Shoffner, J.M. et al. (1989) Proc. Natl. Acad. Sci. U S A 86, 7952-7956
8. Ballinger, S.W. et al. (1992) Nat. Genet. 1, 11-15
9. Ballinger, S.W. et al. (1994) Nat. Genet. 7, 458-459
10. Corral-Debrinski, M. et al. (1991) JAMA 266, 1812-1816
11. Corral-Debrinski, M., Shoffner, J.M., Lott, M.T., and Wallace, D.C. (1992) Mutat. Res. 275, 169-180
12. Corral-Debrinski, M. et al. (1992) Nat. Genet. 2, 324-329
13. Corral-Debrinski, M. et al. (1994) Genomics 23, 471-476
14. Horton, T.M. et al. (1995) Neurology 45, 1879-188
15. Melov, S., Shoffner, J.M., Kaufman, A., and Wallace, D.C. (1995) Nucleic Acids Res. 23, 4122-4126
16. Melov, S., Hinerfeld, D., Esposito, L. and Wallace, D.C. (1997) Nucleic Acids Res. 25, 974-982
17. Michikawa, Y. et al. (1999) Science 286, 774-779
18. Murdock, D.G., Christacos, N.C., and Wallace, D.C. (2000) Nucleic Acids Res. 28, 4350-4355
19. Trounce, I. et al. (2000) Nucleic Acids Res. 28, 2164-2170
20. Levy, S.E. et al. (1999) Transgenic Res. 8, 137-145
21. Sligh, J.E. et al. (2000) Am. J. Hum. Genet. 67, 19 (Abstract 53)
22. Levy, S.E., Chen, Y.-S., Graham, B.H., and Wallace, D.C. (2000) Gene 254, 57-66
23. Graham, B. et al. (1997) Nat. Genet. 16, 226-234
24. Esposito, L.A. et al. (1999) Proc. Natl. Acad. Sci. U S A 96, 4820-4825
25. Heddi, A., Stepien, G., Benke, P.J., and Wallace, D.C. (1999) J. Biol. Chem. 274, 22968-22976
26. Murdock, D., Boone, B.E., Esposito, L., and Wallace, D.C. (1999) J. Biol. Chem. 274, 14429-14433
27. Esposito, L.A. et al. (2000) Free Radic. Biol. Med. 28, 754-766
28. Li, Y. et al. (1995) Nat. Genet. 11, 376-381
29. Melov, S. et al. (1999) Proc. Natl. Acad. Sci. U S A 96, 846-851
30. Melov, S. et al. (2000) Science 289, 1567
